# Prevalence of diabetic nephropathy complicating non-diabetic renal disease among Chinese patients with type 2 diabetes mellitus

**DOI:** 10.1186/2047-783X-18-4

**Published:** 2013-02-22

**Authors:** Li Zhuo, Guming Zou, Wenge Li, Jianhua Lu, Wenwen Ren

**Affiliations:** 1 Department of Nephrology, China-Japan Friendship Hospital, 2 Yinghuayuan East Street, 100029, Beijing, Chaoyang District, China

**Keywords:** Diabetic nephropathy, Non-diabetic renal disease, Diabetes mellitus, Renal biopsy, Renal histopathology

## Abstract

**Background:**

The incidence of diabetes mellitus (DM) and diabetic nephropathy (DN) have risen rapidly in the past few decades and have become an economic burden to the healthcare system in China. DN is a major complication of DM and is a leading cause of end-stage renal disease (ESRD). The occurrence of non-diabetic renal disease (NDRD) in diabetic patients has been increasingly recognized in recent years. It is generally believed that it is difficult to reverse DN, whereas some cases of NDRD are readily treatable and remittable. However, DN is known to co-exist with NDRD in a poorly defined population of patients with type 2 diabetes mellitus (T2DM). This study estimated the prevalence of co-existing DN and NDRD in Chinese patients.

**Methods:**

Data were retrospectively analyzed from 244 patients with T2DM who had undergone a renal biopsy between January 2003 and December 2011 at the Nephrology Department, China-Japan Friendship Hospital, China. Male patients numbered 151 (61.9%) of the study population. The biopsies were performed because urinary abnormalities or renal function were atypical of a diagnosis of DN. Biopsy samples were examined using light, immunofluorescence (IF) and electron microscopy (EM). Clinical parameters were recorded for each patient at the time of biopsy.

**Results:**

Nineteen of 244 diabetic patients (7.8%) had co-existing DN and NDRD. These patients showed clinical features and pathologic characteristics of DN, including a high prevalence of diabetic retinopathy (89.5%), a long duration of diabetes, increased thickness of the glomerular basement membrane (GBM) and mesangial expansion. However, they also presented with clinical findings which were inconsistent with DN, such as hematuria, rapidly progressive renal failure and marked proteinuria. Immunoglobulin A (IgA) nephropathy was apparent in 10 out of the 19 patients (52.6%), tubulointerstitial lesions were found in four patients (21.1%), membrano-proliferative glomerulonephritis (MPGN) in three patients (15.8%) and membranous nephropathy (MN) in two patients (10.5%).

**Conclusion:**

Retrospective analysis of biopsy data suggests that approximately 8% of Chinese patients with T2DM may have co-existing DN and NDRD. The most common histological diagnosis in our small series was IgA nephropathy.

## Background

The incidence of diabetes mellitus (DM) and diabetic nephropathy (DN) have risen rapidly in the past few decades and have become an economic burden to the healthcare system in China. DN, also known as diabetic glomerulosclerosis or diabetic kidney disease, is a major complication of DM and is a leading cause of end-stage renal disease (ESRD). Persistent and slowly progressive proteinuria is a characteristic of DN and diabetic renal failure [1-5]. The diagnosis of DN is usually inferred in cases where renal biopsy has not produced definitive results. These are usually patients with a 7 to 10-year history of type I DM, who have demonstrable diabetic retinopathy and a history of microalbuminuria. These patients present no evidence of a sudden onset of marked proteinuria, hematuria, abnormal kidney size, or other renal disease [5-8]. Renal biopsy in this setting will not be diagnostically useful, while it will be inferred as DN.

Most of our knowledge with respect to the nature of kidney disease in patients with type 2 diabetes mellitus (T2DM) is derived from studies of patients with type I DM [5]. However, biopsy data from patients with T2DM with renal disease or proteinuria show that these patients have a more heterogeneous group of renal lesions than patients with type I DM [3-5,7-9]. Based on these findings the occurrence of non-diabetic renal disease (NDRD) in diabetic patients has been increasingly recognized in recent years. However, the prevalence of NDRD varies widely in different regions of the world and is reported to range from 15.7% to 82.9% [10-27].

It is generally believed that it is difficult to reverse DN, whereas some cases of NDRD are readily treatable and remittable. However, in some diabetic patients, DN and NDRD co-exist. Based on data published between 1983 and 2012 (Table 1), the prevalence of DN complicating NDRD ranges between 3.0% and 45.8% [6,10,12,13,15-17,19,21,22],[28-30].

**Table 1:** Diabetic nephropathy (DN) with non-diabetic renal disease (NDRD) in diabetic patients: literature summary

**Pathological types**	**Number of cases (%)**	**References**
**Glomerular diseases**		
IgA nephropathy	31 (14.1%)	[10,15,16,21,28,30]
Mesangial proliferative glomerulonephritis	22 (10%)	[12,15,19,28,30]
Post-infectious glomerulonephritis	22 (10%)	[12,29,30]
Membranous nephropathy	15 (6.8%)	[6,10,15,17,21,28]
Immune complex-trapping glomerulonephritis	12 (5.5%)	[13,29,30]
Crescentic glomerulonephritis	8 (3.6%)	[12,13,16]
Focal segmental glomerulosclerosis	7 (3.2%)	[10,12,13,15,16]
Minimal change glomerulopathy	3 (1.4%)	[12,19]
Membrano-proliferative glomerulonephritis	3 (1.4%)	[10,16,29]
Lupus glomerulonephritis	2 (1.0%)	[29]
Fibrillary glomerulonephritis	1 (0.5%)	[16]
Necrotizing focal glomerulonephritis	1 (0.5%)	[29]
Hepatitis-related nephritis	1 (0.5%)	[17]
**Vascular**		
Hypertensive changes and arterionephrosclerosis	19 (8.6%)	[12,16,17,22,30]
Atheroembolic renal disease	2 (1.0%)	[12]
Thrombotic microangiopathy	1 (0.5%)	[13]
**Tubulointerstitial**		
Tubulointerstitial nephritis	50 (22.7%)	[6,10,12,16,22,30]
Pyelonephritis	5 (2.3%)	[12,16,22]
Toxemia of pregnancy	1 (0.5%)	[28]
Other	14 (6.4%)	[10,30]
**Total**	**220**	

*DN*, diabetic nephropathy; *IgA*, immunoglobulin A; *NDRD*, non-diabetic renal disease.

The present retrospective, single center, analysis was undertaken to estimate the prevalence of DN complicating NDRD in Chinese patients with T2DM. The pathogenic mechanism of DN that complicates NDRD is thought to be different to the mechanisms that underlie either DN or NDRD alone [10,18,21,22,27]. We, therefore, also studied the correlations between the clinical and pathologic features in these patients.

## Methods

### Patient selection

The study included data from 244 patients with T2DM who underwent renal biopsy between January 2003 and December 2011 at the Nephrology Department, China-Japan Friendship Hospital, China. Male patients numbered 151 (59.4%) of the study population. Patients with known DN were excluded from the study as renal biopsy was not routinely performed in these patients at this institution. Patients with current acute illness including infectious disease were also excluded from entry. The existence of immunologic diseases, malignancy and infections was also investigated prior to inclusion into the study.

The study protocol was approved by the Human Ethics Review Committee of the China-Japan Friendship Hospital and a signed consent form was obtained from each patient before biopsy.

### Assessments

DM was diagnosed using the criteria of the American Diabetes Association [31]. The clinical and demographic parameters were recorded for each patient at the time of renal biopsy. These included duration of diabetes, blood pressure, electrocardiogram, echocardiogram, kidney ultrasound and funduscopic findings. Laboratory studies included fasting blood sugar, glucose tolerance test, glycated hemoglobin (HbA1c), urinalysis, urine osmotic pressure, 24-hour protein excretion, total protein, albumin, blood urea nitrogen, serum creatinine levels and creatinine clearance.

Nephrotic syndrome was defined as proteinuria (>3.5 g/day) accompanied by edema, hyperlipidemia, hypoproteinemia, or other metabolic disorders [32]. Nephritic syndrome was defined as pathologically diffuse inflammatory changes in the glomeruli accompanied by hematuria with red blood cell casts, mild proteinuria, and, often, hypertension, edema and azotemia. Renal failure was diagnosed in patients with blood urea nitrogen >20 mg/dl and serum creatinine >1.4 mg/dl. Hypertension was defined as a systolic blood pressure >140 mmHg or diastolic blood pressure >90 mmHg.

### Biopsy assessment

In all cases renal biopsy was performed because urinary abnormalities or renal function was inconsistent with the clinical expression or the natural history of DN. Percutaneous renal biopsy were performed as described by Veiga [33].

Renal tissue submitted for diagnosis was divided into three portions. One portion was fixed in buffered formalin and processed onto paraffin blocks for light microscopy examination. Sections were stained with hematoxyline and eosin, periodic acid-Schiff (PAS), silver methanamine and Masson’s trichrome. The second portion of tissue was frozen for direct immunofluorescence (IF) studies using fluorescein isothiocyanate (FITC) conjugated antibodies for immunoglobulin A (IgA), immunoglobulin G (IgG), immunoglobulin M (IgM), C3, C1q and fibrinogen (FIB). The third tissue portion was fixed in Trump’s electron microscopy (EM) fixative and processed into resin blocks. Ultrathin sections were stained with uranyl acetate and lead citrate, and examined using a transmission electron microscope.

Renal lesions were classified according to the Chinese classification criteria for renal diseases [34]. Each biopsy was reviewed by two pathologists, both dedicated to renal pathology.

### Statistical analysis

Statistical analysis was performed using SPSS version 17.0 for Windows (IBM SPSS Statistics, Armonk, NY, USA). Data were expressed as mean ±SD. The Spearman rank correlation test was used to determine the associations between clinical parameters and histopathological findings. Values of *P* <0.05 were considered statistically significant.

## Results

Data were retrospectively analyzed from 244 patients with T2DM. Male patients numbered 151 (61.9%) of the study population. There were 20 cases (8.2%) with a pathologic diagnosis of DN, 205 cases (84%) with NDRD and 19 cases (7.8%) had DN complicating NDRD. The proportion of males was 65% in DN, 59% in DNRD and 89.5% in DN complicating NDRD, respectively. The clinical features and laboratory data are summarized in Table 2. The population included 17 men and two women between 28 and 64 years of age, with only one patient older than 60 years. All patients had T2DM; the duration of diabetes at the time of renal biopsy ranged from 2 to 20 years. Diabetic retinopathy was present in 17 patients and the majority of patients had hematuria (15 cases, 78.9%). Fourteen patients had hypertension, four patients had nephrotic syndrome, six patients had nephritic syndrome and nine patients had renal dysfunction.

**Table 2:** The clinical data of diabetic nephropathy (DN) with non-diabetic renal disease (NDRD) in patients with type 2 diabetes mellitus (T2DM)

**Case**	**Age (years)/ sex**	**Duration of T2DM (years)**	**Diabetic retinopathy**	**Fasting blood sugar (mmol/l)**	**HbA1c (%)**	**Clinical manifestations**	**Hematuria**	**Urinary protein (g/day)**	**BUN (mg/dl)**	**Serum creatinine (mg/dl)**	**History of hypertension**
1	41/M	12	+	7.9	7.0	CRF	2+	3.8	23.2	1.5	+
2	31/M	NA	-	6.3	5.4	NG	+	0.9	6.8	1.0	-
3	45/M	7	+	10.6	8.6	NS	2+	3.0	18.9	1.0	+
4	31/M	3	+	4.6	5.7	CRF	-	0.3	20.5	1.5	-
5	53/M	20	+	3.3	5.2	NG	+	6.3	16.9	1.1	+
6	58/M	11	+	7.0	6.5	CRF	+	8.0	62.3	6.9	-
7	64/M	10	+	4.4	7.5	ARF	+	7.0	21.5	3.4	+
8	28/M	5	+	9.0	7.8	NG	+	1.3	13.5	0.6	-
9	48/F	14	+	8.9	4.8	CRF	2+	4.9	21.7	1.4	+
10	39/M	9	+	6.2	5.3	CRF	2+	5.4	29.3	2.5	+
11	58/M	15	+	4.8	6.4	NG	2+	5.6	17.8	1.2	+
12	44/F	16	+	8.3	10.6	NS	-	5.4	23.2	0.7	+
13	54/M	2	+	9.5	7.3	NG	2+	1.7	17.3	1.1	+
14	55/M	13	+	7.2	7.9	NS	2+	10.0	20	1.1	+
15	46/M	15	+	19.2	11.5	CRF	2+	3.2	22.2	1.4	+
16	47/M	5	+	5.1	6.0	NS	+	7.0	17.4	0.9	-
17	54/M	10	+	5.9	6.4	CRF	-	4.4	46.0	2.9	+
18	57/M	2	+	5.0	5.0	ARF	-	10.9	40.2	2.9	+
19	52/M	6	-	5.1	5.6	NG	3+	6.6	42.5	1.3	+

*ARF*, Acute renal failure; *BUN*, Blood urea nitrogen; *CRF*, chronic renal failure; *DN*, diabetic nephropathy; *HbA1c*, glycated hemoglobin; *NA*, not available; *NDRD*, non-diabetic renal disease; *NG*, nephritic syndrome; *NS*, nephrotic syndrome; *T2DM*, type 2 diabetes mellitus.

Patients with a longer history of diabetes had higher urinary protein levels than those with a shorter duration of diabetes (r = 0.67, *P* <0.05). However, there was a poor correlation between disease duration and fasting blood sugar (r = 0.116, *P* >0.05) and HbA1c (r = 0.256, *P* >0.05).

**Figure 1:**
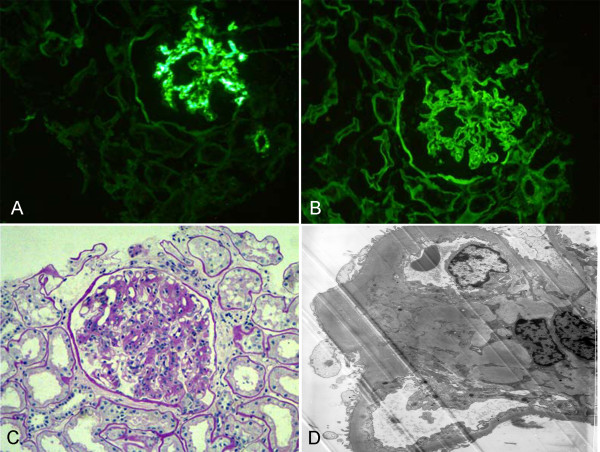
**A case with immunoglobulin A (IgA) nephropathy and diabetic nephropathy (DN).** (**A**) Typical mesangial absorbance pattern after labeling with anti-IgA antibody (IF, 200×). (**B**) The deposits of mainly IgG collected in the basement membrane and appeared in the linear pattern as shown by immunofluorescence (IF, 200×). (**C**) Mesangial cellularity and matrix increased, and there was a thickening of the glomerular basement membrane (GBM) (PAS, 200×). (**D**) The electron micrograph demonstrated an increase in small dense deposits in the mesangium and the mesangial matrix. The basement membrane was diffusely thickened due to diabetic involvement (EM, 5000×). DN, diabetic nephropathy; EM, electron microscopy; GBM, glomerular basement membrane; IF, immunofluorescence; IgA, immunoglobulin A; IgG, immunoglobulin G; PAS, periodic acid-Schiff.

**Figure 2:**
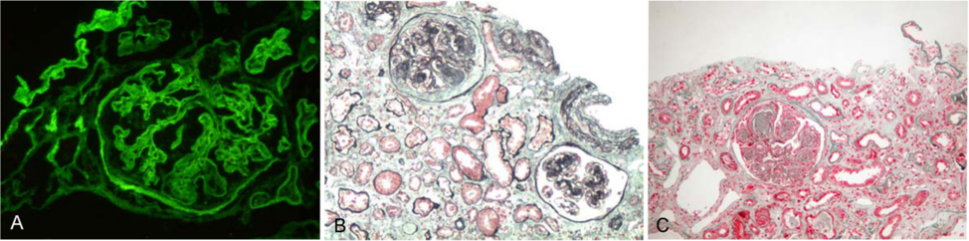
**A case with chronic tubular interstitial nephritis (TIN) and diabetic nephropathy (DN).** (**A**) Deposits of mainly IgG collected in the basement membrane and appeared in a linear pattern as viewed by immunofluorescence (IF, 200×). (**B**) (PAS, 100×) and (**C**) (Masson, 100×) show severe mesangial expansion (Kimmelstiel-Wilson nodules) and severe tubular injury with only minimal cell infiltration in the interstitial area. DN, diabetic nephropathy; IF, immunofluorescence; IgG, immunoglobulin G; PAS, periodic acid-Schiff; TIN, tubular interstitial nephritis.

**Figure 3:**
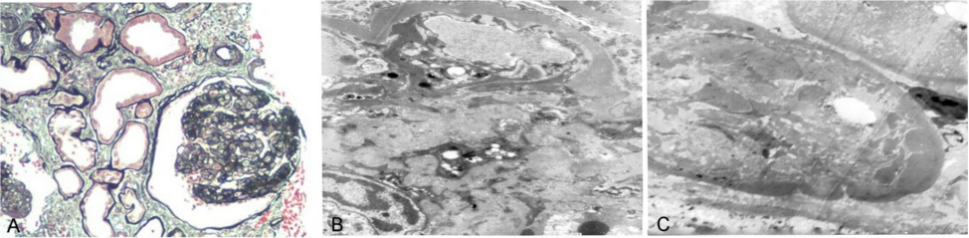
**A case with membrano-proliferative glomerulonephritis (MPGN) and diabetic nephropathy (DN).** (**A**) This silver staining demonstrated a double contour of many basement membranes, with 'tram-tracking', which is characteristic of type I MPGN (PAS, 200×). (**B**) Prominent subendothelial deposits and mesangial interposition are seen (EM, 5000×) and (**C**) the basement membrane was thickened due to diabetic involvement (EM, 5000×). DN, diabetic nephropathy; EM, electron microscopy; MPGN, membrano-proliferative glomerulonephritis; PAS, periodic acid-Schiff.

**Figure 4:**
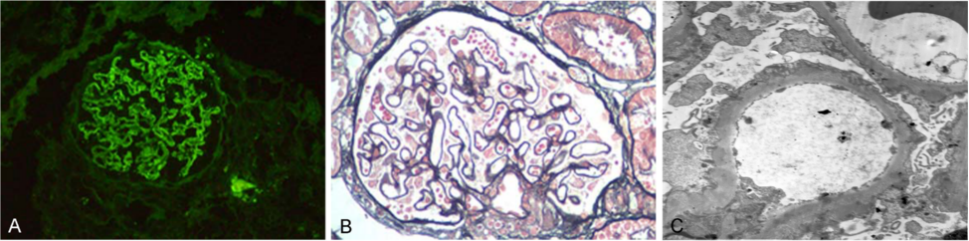
**A case with membranous nephropathy (MN) and diabetic nephropathy (DN).** (**A**) Deposits of mainly IgG collected in the basement membrane and appeared as a diffuse granular pattern as shown by immunofluorescence (IF, 200×). (**B**) Light microscopy showing membranous glomerulonephritis in which the capillary loops were thickened and prominent. Numerous granular dense deposits were located in subepithelial areas (PAS, 200×). (**C**) Thickened glomerular basement membrane (GBM) with numerous granular, dense deposits located in subepithelial areas (EM, 5000×). DN, diabetic nephropathy; GBM, glomerular basement membrane; IF, immunofluorescence; IgG, immunoglobulin G; MN, membranous nephropathy; PAS, periodic acid-Schiff.

Renal pathology findings are summarized in Table 3. All patients had diabetic renal lesions complicated with NDRD. The most common NDRD finding was IgA nephropathy (Figure 1), which was present in 10 patients (52.6%). Four patients had tubulointerstitial lesions (Figure 2), three patients had membrano-proliferative glomerulonephritis (MPGN) (Figure 3) and two patients had membranous nephropathy (MN) (Figure 4).

**Table 3:** The histology of diabetic nephropathy (DN) with non-diabetic renal disease (NDRD)

**Case**	**IgA**	**IgM**	**IgG**	**C3**	**C1q**	**FIB**	**Diabetic renal lesion**	**NDRD**
1	2+	2+	2+	+	±	-	Diffuse and nodular glomerulosclerosis	IgA nephropathy
2	2+	2+	-	2+	-	3+	Diffuse glomerulosclerosis	IgA nephropathy
3	-	-	-	-	-	-	Diffuse and nodular glomerulosclerosis	Membrano-proliferative glomerulonephritis
4	2+	±	+	3+	-	2+	Diffuse and nodular glomerulosclerosis	IgA nephropathy
5	±	3+	2+	3+	2+	-	Diffuse and nodular glomerulosclerosis	Membranous nephropathy
6	±	+	-	-	-	-	Diffuse glomerulosclerosis	Membrano-proliferative glomerulonephritis
7	-	2+	-	3+	+	-	Diffuse and nodular glomerulosclerosis	Tubulointerstitial lesion
8	3+	+	2+	2+	-	2+	Diffuse glomerulosclerosis	IgA nephropathy
9	2+	+	3+	3+	-	±	Diffuse and nodular glomerulosclerosis	IgA nephropathy
10	-	2+	2+	-	-	+	Diffuse and nodular glomerulosclerosis	Tubulointerstitial lesion
11	2+	+	+	±	-	2+	Diffuse and nodular glomerulosclerosis	IgA nephropathy
12	2+	+	2+	3+	-	2+	Diffuse glomerulosclerosis	IgA nephropathy
13	2+	-	2+	-	-	+	Diffuse and nodular glomerulosclerosis	IgA nephropathy
14	2+	2+	+	2+	-	-	Diffuse glomerulosclerosis	IgA nephropathy
15	2+	+	2+	-	-	-	Diffuse and nodular glomerulosclerosis	IgA nephropathy
16	2+	-	3+	3+	-		Diffuse glomerulosclerosis	Membranous nephropathy
17	±	-	2+	-	-	-	Diffuse glomerulosclerosis	Tubulointerstitial lesion
18	-	3+	3+	2+	-	2+	Diffuse and nodular glomerulosclerosis	Tubulointerstitial lesion
19	2+	2+	3+	-	±	-	Diffuse and nodular glomerulosclerosis	Membrano-proliferative glomerulonephritis

The fluorescent staining was graded as follows: - negative, ± doubtful, + weak, 2+ moderate, 3+ bright. DN, diabetic nephropathy; *FIB*, fibrinogen; *IgA*, immunoglobulin A; *IgG*, immunoglobulin G; *IgM*, immunoglobulin M; *NDRD*, non-diabetic renal disease.

## Discussion

The occurrence of NDRD in diabetic patients is well recognized. A variety of renal lesions can occur in diabetic patients, such as IgA nephropathy, MN, mesangial proliferative glomerulonephritis, hypertensive renal disease and focal segmental glomerular sclerosis. In some diabetic patients, DN is complicated by co-existing NDRD. We summarize the relevant studies published between 1983 to 2012 [6,10,12,13,15-17,19,21,22],[28-30], which indicated that glomerular disease (58.2%) remains the most common renal complication in diabetic patients with co-existing DN and NDRD. The review showed that the three most common forms of glomerular disease were IgA nephropathy (14.1%), mesangial proliferative glomerulonephritis (10%) and post-infectious glomerulonephritis (10%). Tubulointerstitial lesions (25.5%) and vascular lesions (10%) also existed in such patients.

In the present study, we found a high incidence of DN complicating NDRD (7.8%). These patients all had pathologic hallmarks of DN, including increased thickness of the glomerular basement membrane (GBM) and mesangial expansion. According to the new classes of glomerular lesions in DN, the degree from light to severe are presented as follows: I. Mild or nonspecific light microscopy changes and electron microscopy-proven glomerular basement membrane thickening. IIa. Mild mesangial expansion. IIb. Severe mesangial expansion. III. Nodular sclerosis (Kimmelstiel-Wilson lesion). IV. Advanced diabetic glomerulosclerosis [35,36]. In our study, most patients’ glomerular lesions in DN were IIb or III.

The most common NDRD in our patients was IgA nephropathy, which accounted for 52.6% of all cases, followed by tubulointerstitial lesion (21.1%) and MPGN (15.8%). The high prevalence of IgA nephropathy was in accordance with findings from the aforementioned literature review, which showed prevalence rates ranging from 7.1% to 44.8% [10,15,16,21,28,30]. The disease spectrum of DN complicating NDRD in China might be thought to be different from that in other parts of the world. Our findings are in accordance with those of other studies, which have reported IgA nephropathy to be the most common NDRD pathology in Asian diabetic patients [7,15,37].

There was a high prevalence (89%) of diabetic retinopathy among our 19 cases of co-existing DN and NDRD. The mean duration of diabetes was more than 7 years, and there was a direct association between duration of diabetes and the severity of proteinuria. The poor correlation between disease duration, fasting blood sugar and HbA1c does not necessarily indicate that DN is the only factor affecting disease progression, since the poor correlation coefficients may have been the consequence of the diversity of other types of NDRD. It is possible, however, that is in the small population. The other surprising finding of this study was the high frequency of hypertension (14 cases, 73.7%), especially as some workers report absence of hypertension in DM as one of the differential diagnostic features of NDRD [28]. These findings indicate that blood pressure control is of primary importance in the prevention of progressive renal disease.

Our results indicate that there are distinct clinical and pathologic features in diabetic patients with DN complicating NDRD. These patients have some of the clinical and pathologic features of DN, which include a high prevalence of diabetic retinopathy, a long duration of diabetes, increased thickness of the GBM and mesangial expansion. Other clinical findings are inconsistent with the natural history of DN, including the presence of hematuria, rapidly progressive renal failure and severe proteinuria. These diverse clinical manifestations might be the direct consequence of a different pathologic diagnosis within the population. IgA nephropathy was the most common histological diagnosis in patients undergoing renal biopsy. However, the pathologic types of NDRD in DN complicating NDRD in diabetic patients are no different to those in non-diabetic patients in previous studies [28].

## Conclusions

In this study, retrospective analysis of biopsy data suggests that approximately 8% of Chinese patients with T2DM may have co-existing DN and NDRD. The most common histological diagnosis in our small series was IgA nephropathy. However, it is single-center study with a small number of patients. Larger, multicenter randomized prospective studies are, therefore, required to confirm these preliminary findings.

## Abbreviations

ARF: Acute renal failure;BUN: Blood urea nitrogen;CRF: Chronic renal failure;DM: Diabetes mellitus;DN: Diabetic nephropathy;EM: Electron microscopy;ESRD: End-stage renal disease;FIB: Fibrinogen;FITC: Fluorescein isothiocyanate;GBM: Glomerular basement membrane;HbA1c: Glycated hemoglobin;IF: Immunofluorescence;IgA: Immunoglobulin A;IgG: Immunoglobulin G;IgM: Immunoglobulin M;MN: Membranous nephropathy;MPGN: Membrano-proliferative glomerulonephritis;NDRD: Non-diabetic renal disease;NG: Nephritic syndrome;NS: Nephrotic syndrome;PAS: Periodic acid-Schiff;T2DM: Type 2 diabetes mellitus;TIN: Tubular interstitial nephritis

## Competing interest

The authors declare that they have no competing interests.

## Authors’ contributions

LZ conceived the study and drafted the manuscript. WL participated in the study design and coordination, and helped draft the manuscript. GZ carried out the renal pathology. JL carried out the immunoassays. WR performed the statistical analysis. All authors read and approved the final manuscript.

## References

[B1] Suzuki Y Ueno M Hayashi H Nishi S Satou H Karasawa R Inn H Suzuki S Maruyama Y Arakawa M A light microscopic study of glomerulosclerosis in Japanese patients with noninsulin-dependent diabetes mellitus: the relationship between clinical and histological features Clin Nephrol 1994 42 3 155 162 7994933

[B2] Ruggenenti P Gambara V Perna A Bertani T Remuzzi G The nephropathy of non-insulin-dependent diabetes: predictors of outcome relative to diverse patterns of renal injury J Am Soc Nephrol 1998 9 12 2336 2343 9848788 10.1681/ASN.V9122336

[B3] Gambara V Mecca G Remuzzi G Bertani T Heterogeneous nature of renal lesions in type II diabetes J Am Soc Nephrol 1993 3 8 1458 1466 8490117 10.1681/ASN.V381458

[B4] Spijkerman AM Dekker JM Nijpels G Adriaanse MC Kostense PJ Ruwaard D Stehouwer CD Bouter LM Heine RJ Microvascular complications at time of diagnosis of type 2 diabetes are similar among diabetic patients detected by targeted screening and patients newly diagnosed in general practice: the hoorn screening study Diabetes Care 2003 26 9 2604 2608 10.2337/diacare.26.9.2604 12941726

[B5] Kramer HJ Nguyen QD Curhan G Hsu CY Renal insufficiency in the absence of albuminuria and retinopathy among adults with type 2 diabetes mellitus JAMA 2003 289 24 3273 3277 10.1001/jama.289.24.3273 12824208

[B6] Lin YL Peng SJ Ferng SH Tzen CY Yang CS Clinical indicators which necessitate renal biopsy in type 2 diabetes mellitus patients with renal disease Int J Clin Pract 2009 63 8 1167 1176 10.1111/j.1742-1241.2008.01753.x 18422591

[B7] Mak SK Gwi E Chan KW Wong PN Lo KY Lee KF Wong AK Clinical predictors of non-diabetic renal disease in patients with non-insulin dependent diabetes mellitus Nephrol Dial Transplant 1997 12 12 2588 2591 10.1093/ndt/12.12.2588 9430856

[B8] Mauer SM Chavers BM Steffes MW Should there be an expanded role for kidney biopsy in the management of patients with type I diabetes? Am J Kidney Dis 1990 16 2 96 100 2382660 10.1016/s0272-6386(12)80561-0

[B9] Tone A Shikata K Matsuda M Usui H Okada S Ogawa D Wada J Makino H Clinical features of non-diabetic renal diseases in patients with type 2 diabetes Diabetes Res Clin Pract 2005 69 3 237 242 10.1016/j.diabres.2005.02.009 16098920

[B10] Chang TI Park JT Kim JK Kim SJ Oh HJ Yoo DE Han SH Yoo TH Kang SW Renal outcomes in patients with type 2 diabetes with or without coexisting non-diabetic renal disease Diabetes Res Clin Pract 2011 92 2 198 204 10.1016/j.diabres.2011.01.017 21320734

[B11] Lee EY Chung CH Choi SO Non-diabetic renal disease in patients with non-insulin dependent diabetes mellitus Yonsei Med J 1999 40 4 321 326 10487133 10.3349/ymj.1999.40.4.321

[B12] Soni SS Gowrishankar S Kishan AG Raman A Non diabetic renal disease in type 2 diabetes mellitus Nephrology (Carlton) 2006 11 6 533 537 10.1111/j.1440-1797.2006.00681.x 17199793

[B13] Nzerue CM Hewan-Lowe K Harvey P Mohammed D Furlong B Oster R Prevalence of non-diabetic renal disease among African-American patients with type II diabetes mellitus Scand J Urol Nephrol 2000 34 5 331 335 10.1080/003655900750048378 11186474

[B14] Jalalah SM Non-diabetic renal disease in diabetic patients Saudi J Kidney Dis Transpl 2008 19 5 813 816 18711304

[B15] Suzuki D Takano H Toyoda M Umezono T Uehara G Sakai T Zhang SY Mori Y Yagame M Endoh M Sakai H Evaluation of renal biopsy samples of patients with diabetic nephropathy Intern Med 2001 40 11 1077 1084 10.2169/internalmedicine.40.1077 11757760

[B16] Ghani AA Al Waheeb S Al Sahow A Hussain N Renal biopsy in patients with type 2 diabetes mellitus: indications and nature of the lesions Ann Saudi Med 2009 29 6 450 453 10.4103/0256-4947.57167 19847082 PMC2881432

[B17] Huang F Yang Q Chen L Tang S Liu W Yu X Renal pathological change in patients with type 2 diabetes is not always diabetic nephropathy: a report of 52 cases Clin Nephrol 2007 67 5 293 297 17542338 10.5414/cnp67293

[B18] Yaqub S Kashif W Hussain SA Non-diabetic renal disease in patients with type-2 diabetes mellitus Saudi J Kidney Dis Transpl 2012 23 5 1000 1007 22982913 10.4103/1319-2442.100882

[B19] Li H Li XW Huang QY Ye WL Duan L Li Y Non-diabetic renal disease in type II diabetes mellitus Zhongguo Yi Xue Ke Xue Yuan Xue Bao 2003 25 1 101 104 12905620

[B20] Zukowska-Szczechowska E Tomaszewski M Renal affection in patients with diabetes mellitus is not always caused by diabetic nephropathy Rocz Akad Med Bialymst 2004 49 185 189 15631340

[B21] Oh SW Kim S Na KY Chae DW Kim S Jin DC Chin HJ Clinical implications of pathologic diagnosis and classification for diabetic nephropathy Diabetes Res Clin Pract 2012 97 3 418 424 10.1016/j.diabres.2012.03.016 22521535

[B22] Chong YB Keng TC Tan LP Ng KP Kong WY Wong CM Cheah PL Looi LM Tan SY Clinical predictors of non-diabetic renal disease and role of renal biopsy in diabetic patients with renal involvement: a single centre review Ren Fail 2012 34 3 323 328 10.3109/0886022X.2011.647302 22250665

[B23] Mou S Wang Q Liu J Che X Zhang M Cao L Zhou W Ni Z Prevalence of non-diabetic renal disease in patients with type 2 diabetes Diabetes Res Clin Pract 2010 87 3 354 359 10.1016/j.diabres.2009.11.012 20005594

[B24] Lu B Gong W Yang Z Yang Z Yang Y Wen J Zhao N Zhu X Hu R An evaluation of the diabetic kidney disease definition in chinese patients diagnosed with type 2 diabetes mellitus J Int Med Res 2009 37 5 1493 1500 19930856 10.1177/147323000903700526

[B25] Hashim Al-Saedi AJ Pathology of nondiabetic glomerular disease among adult Iraqi patients from a single center Saudi J Kidney Dis Transpl 2009 20 5 858 861 19736492

[B26] Chawarnkul O Vareesangthip K Ongajyooth L Cheunsuchon B Parichatikanond P Non-diabetic glomerular disease in type II DM: 10 years experience J Med Assoc Thai 2009 92 Suppl 2 S57 S60 19562987

[B27] Zhuo L Ren W Li W Zou G Lu J Evaluation of renal biopsies in type 2 diabetic patients with kidney disease: a clinicopathological study of 216 cases Int Urol Nephrol 2013 45 1 173 179 10.1007/s11255-012-0164-6 22467137

[B28] Hironaka K Makino H Ikeda S Haramoto T Ota Z Nondiabetic renal disease complicating diabetic nephropathy J Diabet Complications 1991 5 2–3 148 149 1770026 10.1016/0891-6632(91)90051-p

[B29] Kasinath BS Mujais SK Spargo BH Katz AI Nondiabetic renal disease in patients with diabetes mellitus Am J Med 1983 75 4 613 617 10.1016/0002-9343(83)90442-4 6226200

[B30] Pham TT Sim JJ Kujubu DA Liu IL Kumar VA Prevalence of nondiabetic renal disease in diabetic patients Am J Nephrol 2007 27 3 322 328 10.1159/000102598 17495429

[B31] American Diabetes Association Diagnosis and classification of diabetes mellitus Diabetes Care 2007 30 Suppl 1 S42 S47 17192378 10.2337/dc07-S042

[B32] Barry M Brenner BM, Levine SA **The Kidney** Brenner and Rector's the Kidney 2007 Philadelphia: Saunders

[B33] Veiga PA Moxey-Mims MM Springate JE Feld LG A simple method for percutaneous renal biopsy Child Nephrol Urol 1991 11 4 196 198 1777900

[B34] Zou W The guidance of renal pathologic diagnostic criteria Chin J Nephrol 2001 17 1 270 275

[B35] Tervaert TW Mooyaart AL Amann K Cohen AH Cook HT Drachenberg CB Ferrario F Fogo AB Haas M de Heer E Joh K Noël LH Radhakrishnan J Seshan SV Bajema IM Bruijn JA Renal Pathology Society Pathologic classification of diabetic nephropathy J Am Soc Nephrol 2010 21 4 556 563 10.1681/ASN.2010010010 20167701

[B36] Fioretto P Mauer M Diabetic nephropathy: diabetic nephropathy-challenges in pathologic classification Nat Rev Nephrol 2010 6 9 508 510 10.1038/nrneph.2010.96 20736983

[B37] Zhou J Chen X Xie Y Li J Yamanaka N Tong X A differential diagnostic model of diabetic nephropathy and non-diabetic renal diseases Nephrol Dial Transplant 2008 23 6 1940 1945 10.1093/ndt/gfm897 18156459

